# Small number enumeration processes of deaf or hard-of-hearing students: A study using eye tracking and artificial intelligence

**DOI:** 10.3389/fpsyg.2022.909775

**Published:** 2022-08-22

**Authors:** Maike Schindler, Jan H. Doderer, Anna L. Simon, Erik Schaffernicht, Achim J. Lilienthal, Karolin Schäfer

**Affiliations:** ^1^Department of Special Education and Rehabilitation, Faculty of Human Sciences, University of Cologne, Cologne, Germany; ^2^School of Science and Technology, Örebro University, Örebro, Sweden

**Keywords:** mathematics education, deaf or hard-of-hearing students, mathematical difficulties, small number enumeration, eye tracking, Artificial Intelligence

## Abstract

Students who are deaf or hard-of-hearing (DHH) often show significant difficulties in learning mathematics. Previous studies have reported that students who are DHH lag several years behind in their mathematical development compared to hearing students. As possible reasons, limited learning opportunities due to a lesser incidental exposure to numerical ideas, delays in language and speech development, and further idiosyncratic difficulties of students who are DHH are discussed; however, early mathematical skills and their role in mathematical difficulties of students who are DHH are not explored sufficiently. In this study, we investigate whether students who are DHH differ from hearing students in their ability to enumerate small sets (1–9)—an ability that is associated with mathematical difficulties and their emergence. Based on a study with *N* = 63 who are DHH and *N* = 164 hearing students from third to fifth grade attempting 36 tasks, we used eye tracking, the recording of students' eye movements, to qualitatively investigate student enumeration processes. To reduce the effort of qualitative analysis of around 8,000 student enumeration processes (227 students x 36 tasks), we used Artificial Intelligence, in particular, a clustering algorithm, to identify student enumeration processes from the heatmaps of student gaze distributions. Based on the clustering, we found that gaze distributions of students who are DHH and students with normal hearing differed significantly on a group level, indicating differences in enumeration processes, with students who are DHH using advantageous processes (e.g., enumeration “at a glance”) more often than hearing students. The results indicate that students who are DHH do not lag behind in small number enumeration as compared to hearing students but, rather, appear to perform better than their hearing peers in small number enumeration processes, as well as when conceptual knowledge about the part-whole relationship is involved. Our study suggests that the mathematical difficulties of students who are DHH are not related to difficulties in the small number enumeration, which offers interesting perspectives for further research.

## Introduction

Studies throughout recent decades repeatedly indicate that students who are deaf or hard-of-hearing (DHH) tend to have mathematical difficulties, which appear to be severe in many cases (e.g., Traxler, 2000; Blatto-Vallee et al., 2007; Qi and Mitchell, 2012; Marschark et al., 2013; Pagliaro, 2015). Even at preschool age, most students who are DHH show difficulties in their mathematical development (e.g., Kritzer, 2009; Pagliaro and Kritzer, 2013), and these difficulties tend to increase through school age and into adulthood (Bull et al., 2011; Gottardis et al., 2011).

Students with hearing loss can be referred to as DHH, depending on their degree of hearing loss or the hearing threshold (World Health Organization, 2021). A distinction is made between mild, moderate, moderately severe, severe, profound, or complete hearing loss (World Health Organization, 2021). In many cases, both ears are affected. Hearing loss might lead to difficulties in hearing auditory stimuli and communicating with others (Spencer and Marschark, 2010). Moreover, congenital hearing loss has an impact on learning and social development (Knoors and Marschark, 2014). With increased early detection and quality of hearing technology and early intervention, today, many children who were born congenitally deaf can communicate in spoken language due to their hearing technology (Spencer and Marschark, 2010). The increasing importance of bimodal-bilingual education has led to many children today having the opportunity to acquire both spoken and sign language from an early age (Marschark and Knoors, 2012). As a trend, the proportion of students receiving mainstream education has increased in recent years in many countries, and, at the same time, sign language remains an important part of the diverse DHH education (Leigh and Marschark, 2016; Marschark and Leigh, 2016). Despite this, school education for students who are DHH widely varies internationally. Didactical concepts may also differ depending on hearing status, communication modality, and additional needs, both in special schools and in mainstream education.

Mathematical abilities of students who are DHH have received relatively little attention as compared to, for example, their language and literacy skills, which is possibly related to “a pervasive belief that mathematics is ‘not as important' as language and/or literacy” (Pagliaro and Kritzer, 2013, p. 150) among practitioners and researchers working with students who are DHH. Yet, the scarce body of literature on the mathematical abilities of students who are DHH has consistently indicated similar trends: That, they, on average, appear to lag in their mathematical development by several years as compared to their hearing peers. In the 1960s, 1970s, and 1980s, different studies suggested that students who are DHH perform significantly below their same-aged peers with normal hearing (e.g., Wollman, 1965) and that their mathematical development is delayed by 2 to 5 years (Hine, 1970; Wood et al., 1983). These results were validated in studies within the last 30 years, which confirmed that students who are DHH, despite the improvement of early diagnostics, support, hearing technology, educational opportunities, and a greater appreciation of sign language, show a developmental delay of about 3 to 4 years in their mathematical abilities at primary and secondary school levels as compared to their hearing peers (e.g., Heiling, 1995; Frostad, 1996; Traxler, 2000; Qi and Mitchell, 2012; Edwards et al., 2013; Pagliaro, 2015). This discrepancy appears to increase until the age of 16 (Gottardis et al., 2011) and persist even in adulthood (Bull et al., 2011). Taken together, previous studies indicate that students who are DHH tend to have mathematical difficulties that often start as early as preschool age, increase with age, and persist in adulthood. These difficulties apply to a multitude of mathematical topics, including counting (e.g., Pagliaro and Kritzer, 2013), measurement (e.g., Austin, 1975; Pagliaro and Kritzer, 2013), fractions (e.g., Titus, 1995; Bull, 2008; Mousley and Kelly, 2018), number line estimation (e.g., Bull et al., 2011; Bedoya-Ríos and Dorneles, 2021), and word problems (e.g., Kelly and Mousley, 2001; Nunes and Moreno, 2002; Hyde et al., 2003; Ansell and Pagliaro, 2006; Blatto-Vallee et al., 2007). One of the relative strengths of students who are DHH lies in the area of geometry (Pagliaro and Kritzer, 2013), which is presumably related to strong visual and spatial skills, wherein students who are DHH are assumed to have an advantage (e.g., visuospatial working memory, Marschark and Knoors, 2012). For example, children who are DHH were found to be able to remember complex figures better than hearing students, yet, if the figures move and the sequences of movements are then to be described and classified in temporal order, students who are DHH show an inferior performance compared to hearing students (Todman and Seedhouse, 1994; Marschark and Knoors, 2012).

Mathematical delays of students who are DHH appear to start before schooling. When school begins, there seems to be a quantitative delay of about 2 years in mathematical abilities of children who are DHH as compared to hearing children (Swanwick et al., 2005; Pagliaro and Kritzer, 2013). Studies by Kritzer (2009) and Pagliaro and Kritzer (2013) have furthermore indicated that even at preschool age a majority of children who are DHH showed delays in early mathematics, for example, in number comparisons, counting by groups, and word problems (Kritzer, 2009; Pagliaro and Kritzer, 2013). Leybaert and Van Custem (2002) found in preschoolers that “deaf children exhibited age-related lags in their knowledge of the number sequence; they made different errors from those of hearing children, reflecting the rule-bound nature of sign language. Remarkably, their performance in object counting and creating sets of given cardinalities was similar to that of hearing children” (p. 482). Furthermore, Zarfaty et al. (2004) found that preschool children who are deaf and using sign language did not have difficulties in representing and discriminating numbers and that they performed better in spatial (not temporal) tasks than their hearing peers. Yet, it is important to consider that many studies investigated deaf native sign language users and their understanding of number concepts. Even though it is not entirely clear how children who are DHH with hearing parents use sign language, or how children who are DHH use spoken language perform, it was found that better sign language skills of students who are DHH tend to result in better mathematical performance (e.g., Henner et al., 2021). Taken together, the results on the mathematics performance of preschoolers are diverse, indicating that it is not quite easy to say where the mathematical difficulties of students who are DHH originate, yet certain delays appear to be present even before the school age.

What are the reasons for the difficulties in mathematics of students who are DHH, which appear to start in preschool? One obvious reason may be a delay in language development, which affects many children who are DHH (Spencer and Marschark, 2010). The development of language is closely linked to the development of mathematical thinking and is acquired incidentally and intuitively in everyday life (Nunes and Moreno, 2002). Social activities, such as counting, are learned and taught through language. In this context, children's access to mathematical knowledge is initially informal, without the content being explicitly labeled as mathematical. For example, many nursery rhymes contain counting activities, shape comparisons, or spatial descriptions that are being automated through frequent singing and, later, make it easier for children to acquire counting and mathematical problem-solving skills. Several other factors have been suggested to explain why students who are DHH lag in their mathematical development, which can be broadly divided into two categories: One possible explanation is that the reason lies in fewer learning opportunities for DHH for students that are related to limited incidental exposure to numerical ideas, language issues, and barriers, parents making few references to mathematical concepts in everyday activities, and quality of education (Titus, 1995; Nunes, 2004; Kritzer, 2008, 2009; Barbosa, 2014); the other explanation assumes that the problems originate from students who are DHH themselves, and idiosyncratic difficulties of students who are DHH in basic numerical processing and retrieval processes (e.g., for a detailed discussion, see Zarfaty et al., 2004; Bull et al., 2006; Pagliaro and Kritzer, 2013). In a review of the literature, Santos and Cordes (2022) found that in addition to limited or reduced language access, especially in young children, factors, such as executive functions, specifically, working memory, may also be decisive for the fact that students who are DHH may have difficulties in mathematics. Some previous studies have investigated the basic numerical processing of students who are DHH, in particular. For example, Bull et al. (2006) found that university students who are DHH did not differ from their hearing peers in response times in small-number enumeration processes, indicating that these processes appear not to differ for students who are DHH at the university level. However, the participants in this study were at a high academic level. It is not yet clear whether small number enumeration processes of school-age students who are DHH differ from those of their hearing peers and whether this may be one of the causes for their mathematical difficulties, or whether the difficulties, instead, originate from other factors.

Mathematical difficulties in hearing children are often associated with issues in basic numerical processing (e.g., Butterworth, 2005; Wilson and Dehaene, 2007), and such basic numerical difficulties tend to cascade to severe difficulties in a wide range of mathematical abilities (Butterworth, 2005; Geary, 2013). One of such abilities known to predict mathematical achievement in later school years is a small-number enumeration, the ability to grasp sets of items and say how many there are (Starkey and Cooper, 1995). For children at preschool level and at the beginning of primary school, it is crucial to learn to enumerate quantities (that is, to perceive sets of items and say how many there are) (e.g., Department of Education, 2013). The ability to enumerate small sets of items involves different processes: (perceptual) subitizing, counting, and conceptual subitizing, so-called groupitizing (Gelman and Gallistel, 1986; Schleifer and Landerl, 2011; Ashkenazi et al., 2013; Starkey and McCandliss, 2014). Subitizing means the ability to enumerate small quantities without counting in a fast and exact way; the process is perceptual, automatized, and often subconscious (Clements, 1999; Fischer et al., 2008). Humans—even young children—are usually capable of perceiving quantities of up to four items through subitizing (Starkey and Cooper, 1980, 1995; Mandler and Shebo, 1982; Clarke et al., 2006; Schleifer and Landerl, 2011). Subitizing is a foundation for the development of the concept of numbers and arithmetic learning (Starkey and Cooper, 1995). Humans cannot (in most cases) subitize sets of more than four items; larger numbers of items need to be perceived serially, for example, through counting or structuring them in groups (that can again be subitized). Counting is a process that is socially taught, and it involves conventions that have been summarized by Gelman and Gallistel (1986) as counting principles: *the one-to-one principle*, meaning that each item is assigned exactly with one number word, *the stable-order-principle*, meaning that the order of number words is predetermined (1, 2, 3, 4, …), *the cardinal principle*, meaning that the number word that was said last indicates the cardinality of the set, *the abstraction principle*, meaning that different kinds of objects can be counted, irrespective of their appearance, and *the order-irrelevance principle*, meaning that the order, in which the items are counted, is irrelevant for the cardinality of the set. Compared to subitizing, counting is a “slower and more error-prone process of one-to-one mapping between a set of objects and number words” (Schleifer and Landerl, 2011, p. 280). In the context of studies addressing enumeration processes, researchers refer to the counting range when sets of five or more items are to be enumerated, and to the subitizing range for sets of one to four items, even though, of course, counting processes can also be involved in the enumeration of sets within the subitizing range (e.g., Schleifer and Landerl, 2011; Ashkenazi et al., 2013; Schindler et al., 2020b). Despite subitizing and counting processes, in enumeration, processes of perceiving sets in subsets, dividing sets, and combining subsets to a set are involved (Clements, 1999). This involves the part-whole schema (Starkey and McCandliss, 2014), which means “understanding that quantities can be decomposed into pieces and reassembled again” (Krajewski and Schneider, 2009, p. 513). The enumeration of sets in subsets (groups) is called groupitizing (Starkey and McCandliss, 2014) or “conceptual subitizing” (Clements, 1999) since conceptual processes of the part-whole relationship are involved. Groupitizing allows sets of items to be perceived quasi-simultaneously, that is, by subitizing subsets (Anobile et al., 2020; Wege et al., 2022). Furthermore, enumeration of small sets can involve the recall of familiar patterns, such as dice patterns. In this case, researchers talk about canonical representations (e.g., Ashkenazi et al., 2013). Such patterns do not necessarily have to be counted, but they can be recalled if the image of the pattern is familiar, and, when recognized, the according number can be recalled (e.g., Starkey and McCandliss, 2014). In this case, the child does not need to perceive items serially but can associate the pattern to a number word (Von Glasersfeld, 1982). In general, the canonical representations are symmetrical and, thus, allow the use of patterns or symmetries for number enumeration (Hsin et al., 2021).

Enumeration of small sets is essential for learning basic arithmetic and, therefore, crucial to the development of students' mathematical skills (Starkey and Cooper, 1995). For example, preschoolers' mastery of structured sets (e.g., dice patterns) can predict their arithmetic abilities in grade 1 (Kreilinger et al., 2021), and their subitizing ability can predict their arithmetic performance in later school years (Hannula-Sormunen et al., 2015). Mathematical difficulties (MD)—that is, difficulties in understanding basic arithmetic concepts (e.g., Scherer et al., 2016; Moser Opitz et al., 2017)—are associated (among other factors) with deficits in subitizing that affect enumeration processes (e.g., Butterworth, 2005; Wilson and Dehaene, 2007). Previous research on students' enumeration processes indicates a “dysfunctional subitizing mechanism” (e.g., Schleifer and Landerl, 2011, p. 280) for students with MD (Van der Sluis et al., 2004; Schleifer and Landerl, 2011; Landerl, 2013). Studies addressing, among other things, the students' response times in the enumeration of small sets found that students with MD (aged 7–17, Fischer et al., 2008; aged 10, Moeller et al., 2009) were slower than students without MD in the subitizing range. When examining the enumeration abilities of students in second to fourth grade, students with MD were found to have subitizing problems, specifically, steeper response times slopes (Schleifer and Landerl, 2011; Landerl, 2013). Response time slopes in the counting range were similar for students with and without MD (Schleifer and Landerl, 2011). Further, Van der Sluis et al. (2004) found that fourth to fifth graders with low mathematical skills needed more time for enumeration of sets in the subitizing range. Gray and Reeve (2014) found that weak subitizing profiles of preschoolers were related to poor arithmetic (addition) skills, providing another indicator of difficulties in subitizing for children with MD in their study. Ashkenazi et al. (2013), in a study distinguishing the arrangement of items (canonical vs. random), found that students with MD had higher error rates than the control group when enumerating canonically arranged items, which increased as the number of items increased. For canonically arranged item patterns, longer response times in a group of students with MD—as compared to a control group—were also found by Schindler et al. (2020b). This suggests that students with MD benefit less from canonical arrangements of items (Ashkenazi et al., 2013; Schindler et al., 2020b). Based on product-related data, such as response times or error rates, causes of differences between performances of children with and without MD cannot be definitively explained (Van der Sluis et al., 2004).

However, deficits in subitizing, which may be involved in the emergence of MD, can be further investigated by the analysis of eye movements during enumeration processes (see Mock et al., 2016). To find out whether students with MD are only slower at subitizing or whether they rely on qualitatively different enumeration processes, eye-tracking studies (Moeller et al., 2009; Schindler et al., 2020b) have proven to provide insights into students' enumeration processes. In a case study with students of the same age as the students in this study, Moeller et al. (2009) investigated enumeration processes for small sets of 1 to 8 items. They found differences between children with and without MD, in that counting processes were observed in both students with MD. Another ET study (*N*=20) by Schindler et al. (2020b) also showed differences in enumeration processes between students with and without MD. In both the subitizing (2–4) and the counting (5–9) range, processes of counting all items were observed more frequently for students with MD as compared to their peers without MD, who used (quasi-) simultaneous enumeration more often (Schindler et al., 2020b). These results were also found for the enumeration of items in canonical arrangements (2–9). In summary, qualitatively different processes between students with and without MD were indicated.

This article intends to contribute to the investigation of possible roots of mathematical difficulties of students who are DHH. Specifically, we want to explore if the mathematical difficulties of students who are DHH go along with deficits in small-number enumeration. This study aims to investigate if students who are DHH differ from hearing students in small-number enumeration processes. We ask the research question: “*Do DHH students differ from hearing students in small number enumeration processes?”* To investigate enumeration processes, we conducted a study using eye tracking (ET), the recording of students' eye movements, since ET has shown to be a valuable tool for investigating small number enumeration processes (Moeller et al., 2009; Schindler et al., 2020b). In a study with *N* = 227 students (164 hearing students, 63 students who were DHH), we analyzed eye movements of approximately 8,000 students' enumeration processes (227 students x 36 tasks) and—to reduce the effort of qualitative analysis in a large-scale study with several thousand items—combined it with Artificial Intelligence (AI) to identify student enumeration processes from the heatmaps of student gaze distributions.

With the focus on basic numerical difficulties as a possible root of MD, this paper addresses an issue that is not only of concern for researchers and educators in the domain of students who are DHH and their teaching but beyond: Addressing enumeration, specifically, counting principles or the part-whole schema early on is of concern for many students—for students with mathematical difficulties and students in the domain of special education (e.g., Krajewski and Schneider, 2009; Hecht et al., 2011; Schleifer and Landerl, 2011; Garrote et al., 2015). The diagnostics and support of students with difficulties in these aspects are of paramount importance since longitudinal studies indicate that students who enter school with low early-math skills often do not overcome their deficits during primary school (e.g., Viesel-Nordmeyer et al., 2019). Our study contributes to the research body on student enumerations processes and can be a springboard for further research on students with MD and/or special educational needs.

## Materials and methods

### Participants

A total of *N* = 227 students participated in this study (see Table 1). The sample consisted of a group of hearing students (*n* = 164) and a group of students who are DHH (*n* = 63). The hearing group consisted of students in fifth grade at a German comprehensive school in North Rhine-Westphalia (NRW) aged 9.10 to 12.6 years (mean: 10.9 years, SD: 0.7 years). The group of students who are DHH consisted of fifth-, fourth-, and third-grade students between 9.1 and 13.6 years (mean: 11.9 years, SD: 1) from German special schools for students who are DHH in NRW. The inclusion of students from third and fourth grades in the DHH group served the purpose of approximating the mean age of the participants in the two groups. This appeared to be beneficial, since most of the students who are DHH were in the same grade level (fifth grade) but in a higher school year chronologically than the hearing students. Furthermore, it allowed for a larger sample to be used.

**Table 1:** Participants in the study.

	**Hearing group** **(*n* = 164)**	**DHH group** **(*n* = 63)**
**Participant information**		
Age: mean (standard deviation)	10.9 (0.7)	11.9 (1.0)
Gender: n_girls_ (%_girls_)	72 (43.91)	33 (52.38)
Grade (s): grade (%)	Grade 5 (100)	Grade 3 (6.35)
		Grade 4 (33.33)
		Grade 5 (60.32)
**Mathematical abilities**		
HRT Mean t-score (standard deviation)	40.37 (9.90)	35.04 (9.90)
Mathematical difficulties: *n* (%)	69 (42.07)	41 (65.08)
At risk zone: *n* (%)	36 (21.95)	9 (14.29)
Typically developing: *n* (%)	59 (35.98)	13 (20.63)

The degree of hearing loss in the group of children who are DHH varied, including mild (*n* = 1; 1.6%), moderate (*n* = 19; 30.2%), severe (*n* = 20; 31.7%), and profound (*n* = 21; 33.3%). Two students had a central auditory processing disorder (CAPD) (3.2%), and two other children were affected by single-sided deafness (SSD) (3.2%). Half of the students were fitted with bilateral hearing aids (32; 50.8%), and about a quarter had bilateral cochlear implants (CI) (15; 23.8%). Six students had a bimodal fitting with a hearing aid and a cochlear implant (9.5%). Three students were fitted with a unilateral cochlear implant (4.8%) and one had a unilateral hearing aid (1.5%). One student was fitted with bilateral bone-anchored hearing aids (BAHA) (1.5%). Five of the students, including the two with CAPD, were unaided. As for the students' familial first language (L1), 30 of the students had German as their first language, ten students were bilingual with German and another language (Arabic, Farsi, Italian, Russian, Serbian, Turkish, and Ukrainian), five had Turkish as first language, three Arabic, three German Sign Language (DGS), three Kurdish, and each one Afghan, Greek, Hungarian, Polish, Romanian, Syrian, Tamil, and Urdu, and one that is not specified. While conducting our study, we asked the students for their individual preferred communication modality: 28 of the students preferred spoken language as a communication modality (44.4%), 30 preferred sign-supported speech (47.6%), while five students preferred sign language (7.9%). Sign language, in this case, DGS, is based on a visual-spatial modality and has its own grammatical structure and syntax. While sign language is a language of its own, sign-supported speech only makes use of some of the DGS' signs to support spoken language while speaking. The diversity of the participants who are DHH in this study—especially about prior language experience, communication modality, hearing status, and hearing care—represents well the heterogeneity often encountered in special education schools for children who are DHH in Germany. We chose to include students who attended schools for students who are DHH, who each had a special educational need in hearing, with the commonality of all children in the sample having a peripheral hearing loss and, in a few cases, a central auditory processing disorder, and we refrained from deliberately selecting children that fulfill a specific language or modality profile.

Before the study, legal guardians were informed about the aim and the procedures of the study and that students' participation was voluntary. The participating students were then informed individually about the content and procedures of the study; they had the opportunity to ask questions and to stop the study at their convenience. All students could choose what modality they preferred during the study (spoken language, sign-supported speech, or sign language). The interviewer, who conducted the study, was fluent in all three modalities. It was ensured that all students understood the tasks and felt comfortable during the study.

Prior to the ET study, a standardized arithmetic test, HRT (Haffner et al., 2005), was administered. The HRT is widely used in German-speaking countries to diagnose MD, and it has been used in previous studies examining small number enumeration (e.g., Moeller et al., 2009; Schleifer and Landerl, 2011; Schindler et al., 2019a, 2020b). HRT uses the percentile rank (PR) to diagnose MD, which provides information on what percentage of the norm sample performed equally well or worse (Haffner et al., 2005). If the PR is ≤10, the child is considered to have MD. Between PR 11 to 25, students are within an “at-risk zone” for MD. Students with PR>25 are considered typically developing. In this study, only the first half of the HRT, which focuses on numbers and arithmetic, was administered to the students (similar to Schleifer and Landerl, 2011; Landerl, 2013; Schindler et al., 2019a, 2020b). This arithmetic part alone can be used to diagnose MD (Haffner et al., 2005). The HRT is standardized from the end of first grade through the first six weeks of fifth grade. The sample of students with DHH in this study was tested toward the end of the school year. To use the HRT for fifth graders with DHH at the end of the school year, we adjusted the t-scores following a study by Pitters (2018) that also took place toward the end of the fifth-grade year. Following the average increase in t-scores of 5.5 from the beginning of grade 5 to the end of grade 5 found in Pitters' study, we decreased the t-scores of DHH fifth graders by 5.5 to adjust the values with respect to the standardization at the beginning of fifth grade.

### Eye tracking as a research method in mathematics education

The human eye provides high resolution only in the small region of the fovea, the *fovea centralis* (Holmqvist et al., 2011). This region corresponds to a very small part of our field of view (only about the size of a thumbnail at arms' length). Hence, the eyes need to move, so that the foveal area is oriented toward those regions in the environment from which detailed information is needed (Rayner, 1998; Henderson, 2003). Conversely, following the sequence of movements of the eye allows insight into visual attention and what cognitive processes were associated with the need to collect high-resolution visual information in the observed gaze pattern. Thus, ET, the recording of eye movements that brings areas of interest into the foveal vision, is used as a tool to study cognitive processes in many areas of research, including mathematics education research. ET investigates overt attention since people pay overt attention to those areas that they perceive by the foveal vision: When the eyes move directly to the stimulus, their attention is there, whereas those areas that are perceived peripherally may be taken in by covert attention (Posner, 1980; Carrasco, 2011).

ET is of growing interest in research in mathematics education (and, prospectively, also for educators) as a comparatively unobtrusive tool that allows access even to unconscious cognitive processes (Lilienthal and Schindler, 2019; Strohmaier et al., 2020; Schindler, 2021). Interest in ET has increased since ET devices became more affordable, easy to use, advanced, and accurate (Lilienthal and Schindler, 2019). Another reason for the increasing popularity of ET is that the inherent difficulty to draw conclusive inferences from gaze tracks, as well as the difficulty to extract useful information from a large amount of data, are mitigated by theoretical advances in interpretation (Schindler and Lilienthal, 2019). The interpretation of ET data is often based on the so-called eye-mind hypothesis (Just and Carpenter, 1980). This hypothesis states that there is no significant difference between what the eyes fixate on and what is processed (Holmqvist et al., 2011). Even though this hypothesis, which was developed in reading research (Just and Carpenter, 1980), does not generally hold true in mathematics (see Schindler and Lilienthal, 2019), previous research has shown that the interpretation of eye-tracking data in the domain of enumeration and quantity recognition is more straightforward (Schindler and Lilienthal, 2018; Schindler et al., 2019a, 2020b). Another reason for the growing interest in ET in mathematics education research is that computational resources for automated analysis of gaze data are available at a low cost. This and the other factors mentioned above have opened the prospect of novel applications of ET in mathematics education that use AI for automated or partially automated analysis of gaze data (Schindler et al., 2020a). During the last decade, mathematics education research has seen a notable increase in the number of publications that use ET (Lilienthal and Schindler, 2019; Strohmaier et al., 2020). An extensive review paper by Strohmaier et al. (2020) identified a large variety of topics, including studies of numbers and arithmetic (the largest group), learning difficulties, computer-supported learning, and studies of affective variables (Strohmaier et al., 2020).

### Eye-tracking device and stimuli

We recorded eye movements with the screen-based eye tracker, Tobii Pro X3-120. This eye tracker allows the tracking of eye movements (binocular) with a sampling rate of 120 Hz. It is an eye-tracker shaped like a bar that can be mounted to the bottom of a screen's frame. Therefore, it is unobtrusive and not distracting to participants. Tobii Pro X3-120 uses infrared illuminators to create corneal reflection patterns from participants' eyes. According to the manufacturer, the accuracy is 0.4° (Tobii, 2019). The average accuracy in this study was 0.9°. Participants can move during the recording without affecting accuracy and precision. A 9-point calibration was performed before each data collection.

**Figure 1:**
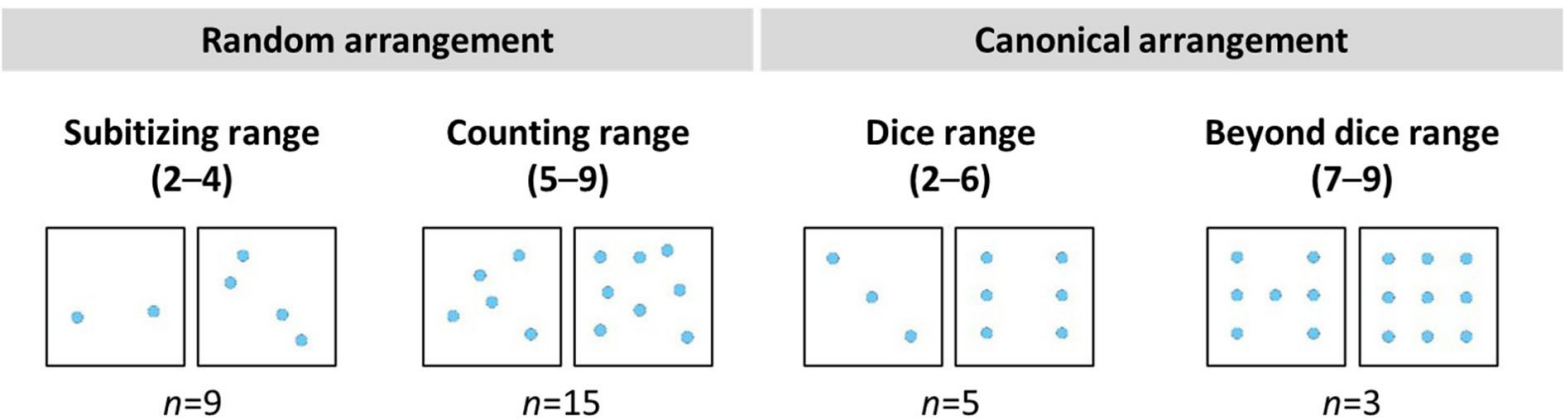
Examples of the items.

In total, there were 36 items representing the sets from 1 to 9. Every quantity was presented in four different arrangements: The sets were each presented in a canonical arrangement and in three random arrangements, arranged differently each time (see Figure 1 for examples). For the random arrangements, two ranges can be distinguished: the subitizing range (2–4) and the counting range (5–9). The canonical arrangements were divided into the dice range (2–6) and beyond the dice range (7–9). The items were the same as in the study by Schindler et al. (2019b).

The enumeration tasks were presented on a 24” full HD screen with a refresh rate of 60 Hz and a resolution of 1,920 x 1,080 pixels. The dots on this screen had a diameter of 2 cm and a maximum span of the dots (vertical/horizontal) of 15 cm. The students sat on a chair about 60 cm from the monitor.

### Machine learning to support the analysis of eye-tracking data

Our study uses ET in combination with machine learning (ML) to investigate student enumeration processes. Generally, the term ML on the one hand refers to a *subject of study* in AI: the study of computer algorithms that can improve automatically through experience and by using training data (Mitchell, 1997). In addition, ML refers to a specific *set of methods* “that can automatically detect patterns in data, and then use the uncovered patterns to predict future data, or to perform other kinds of decision making under uncertainty” (Murphy, 2012, p. 1). It is important to emphasize that often, no explicit manual coding is required. This goes along with one reason why ML is appealing: It promises to shorten or eliminate the laborious process of developing complex software. More importantly, ML allows learning models from examples, which represent patterns or dependencies that are otherwise unknown and cannot be coded analytically.

Among the three major types of ML, supervised learning, unsupervised learning, and reinforcement learning, only supervised and unsupervised machine learning have been used so far in mathematics education, to the best of our knowledge. In this work, we use unsupervised learning. For a better understanding, we describe the general differences between supervised learning and unsupervised learning in this section and discuss related work that used unsupervised learning in mathematics education in the following. In predictive or supervised learning, the goal is to learn a mapping from inputs x to outputs y, given a labeled training set. After training, the learned mapping can be used to make categorical or nominal predictions (Murphy, 2012). Supervised learning (SL) was used, for example, in Schindler et al. (2019b), where the inputs **x** are (as in this paper) the heatmaps that represent the non-temporal information in ET sequences and the labels for each heatmap specify whether it belongs to a student with MD or a student who is typically developing. After training as described in Schindler et al. (2019b), the SL algorithm can classify unseen heatmaps and can be used to predict whether the corresponding student is likely to have MD or not. The second type of ML that has been previously used in mathematics education research is the descriptive or unsupervised learning approach (USL), where training samples **x** but no labels y are given. The computer is then tasked to “find ‘interesting patterns' in the data” (Murphy, 2012, p. 2). This is also called “exploratory data analysis” or “knowledge discovery.” As Murphy (2012) notes, USL is a much less well-defined problem than SL. This is because there is no a-priori guidance about what kinds of patterns to look for, and there is no obvious error metric to use, unlike SL, where a prediction y for a given **x** can be compared to the observed value (Xu and Wunsch, 2009). USL algorithms typically separate the unlabeled training data {**x**} into a number of meaningful clusters, and the major interest in USL is in such clustering algorithms. Clustering can be useful for several reasons, such as to get a compressed representation of the data or to generate hypotheses through data exploration (Aldenderfer and Blashfield, 1984).

In the context of mathematics education, Schindler et al. (2020a) have explored the possibility of identifying student strategies in whole number representations using ET combined with USL. Quantity recognition tasks on the 100-dot field were presented, and, as in this paper, heatmaps were used as representations of gaze sequences. The result of a specific clustering algorithm, Self-Organizing Maps (SOMs, Kohonen, 2001), was then evaluated to find out whether the clusters were consistent with respect to enumeration strategies. The initial results were mixed. The clusters found with the SOM algorithm were consistent to some extent but sometimes also subsumed different strategies in one cluster. This result is not surprising as the clustering used only the visual similarity of heatmaps and discarded, for example, temporal information and information about absolute durations. Other clustering algorithms (for example, with tailored proximity metrics and an optimized intermediate representation) may improve well the consistency. However, an important aspect of Schindler et al. (2020a) study is that clustering was used to provide an independent, non-human view of the data. In this sense, clustering can be seen as an example, in which AI is used to support human researchers—similar to a researcher colleague who takes an independent look at the data with a different perspective. In the future, we can imagine that different USL components, based on different clustering algorithms, generate different category hypotheses and, like an “AI colleague,” make suggestions about meaningful ways to categorize the data. Human researchers would then interpret and verify these suggestions based on pre-studies with smaller numbers of participants and a principled understanding of the applied USL algorithms.

### Procedure

The study took place in individual sessions in a quiet room at the respective schools of the students. Tasks were presented on the full HD screen with the eye tracker attached to the bottom frame. The study began with the calibration procedure, which was followed by three practice tasks to verify and ensure task comprehension. This was followed by 36 enumeration tasks. Participants were instructed to correctly name the number of dots represented as quickly as possible in their preferred communication modality (spoken language, sign-supported speech, or sign language). A fixation star was displayed between tasks in the center of a white screen to separate each task: Students were instructed to fixate on the star as long as it was visible so that students' gazes in the tasks always started from the very same position, the center of the screen. The items were intermixed randomly, using the same order for each student. The students were not given feedback on their responses. Students' responses were recorded with an audio recorder to analyze if they answered correctly (for example, “six” for six dots) or incorrectly. For students who communicated in sign language, responses were recorded in writing. For the analyses of student enumeration processes, the sets with one dot were not included since the enumeration processes can only be meaningfully differentiated from 2 points upwards. Thus, 32 tasks per student were analyzed, resulting in a total of 7,264 tasks. To analyze student enumeration processes, we used heatmaps provided by the Tobii Pro Lab software together with AI (ML).

### Data analysis

#### Input data

**Figure 2:**
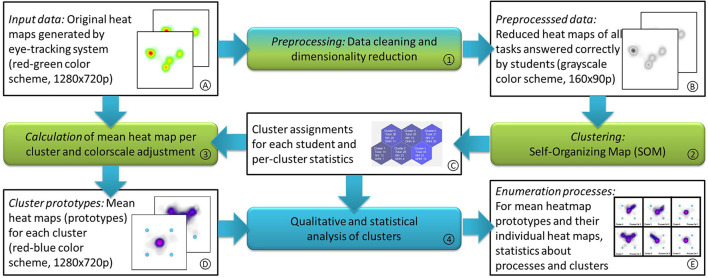
Data flow diagram illustrating the data analysis steps employed in this study. The green processing elements describe steps the system performs automatically, while the turquoise element indicates manual analysis. Rectangular boxes contain raw or processed data. Numerals indicate the order of processing steps; letters indicate the order the data are generated in; and they are referenced in the detailed explanations below.

ET devices, like the one used in this study, offer different representations for the recorded ET data, such as detailed scan paths comprised of saccades and fixation times. A slightly less complex but rather intuitive representation is an aggregated heatmap, where the spatial distribution of gaze targets is represented as an image. This representation loses parts of the temporal information completely, such as the order in which the gaze shifts; and it represents other aspects only in relative terms, such as the time the gaze focuses on a single position. This information is color-coded relative to the length of the complete trial. Despite these shortcomings, patterns are easily accessible for human experts to interpret on heatmaps, and they allow the use of tried and tested image processing methods and ML approaches. Furthermore, previous studies (Schindler et al., 2019a,b) indicate the usefulness of heatmaps for assessing the students' enumeration processes since the enumeration process employed by the student can be identified by a human expert (Schindler et al., 2020a). In our processing pipeline, which is shown in Figure 2, we use the heatmaps generated by the Tobii Pro Lab Software as input data (Figure 2A). Each task of each student generates a single heatmap of 1,920x760 color pixels (~1.5 megapixels) for a total of 7,264 heatmaps with more than 10 billion data points (10,930,867,200).

#### Preprocessing

In an initial data cleaning step, heatmaps were removed from the data pool if the student did not solve the enumeration task correctly (Figure 2.1). This step is necessary to exclude a random gaze pattern where the student just guessed instead of enumerating the dots shown. After performing this filter step, 6,615 usable heatmaps remained.

Each heatmap was reduced in size by converting them to grayscale images and subsampling them to 160 x 90 pixels each (Figure 2B). This drastic reduction in dimensionality (by a factor of more than 300) without losing important information is possible since the heatmap images do not contain high-frequency components (that is, details on the level of a few pixels). Even isolated fixations are represented as smooth blobs, and the minimum blob size is large enough to always be conserved in the reduced-size images. This dimensionality reduction was done for computational reasons only. Executing the clustering algorithm on full-size images will yield very similar if not identical cluster assignments, but the time required on off-the-shelf computer hardware increases significantly: less than an hour to process all tasks with dimensionality-reduced images (160 x 90 gray value pixels) and an estimated week for original-sized images (1920 x 760 color pixels).

#### Clustering

**Figure 3:**
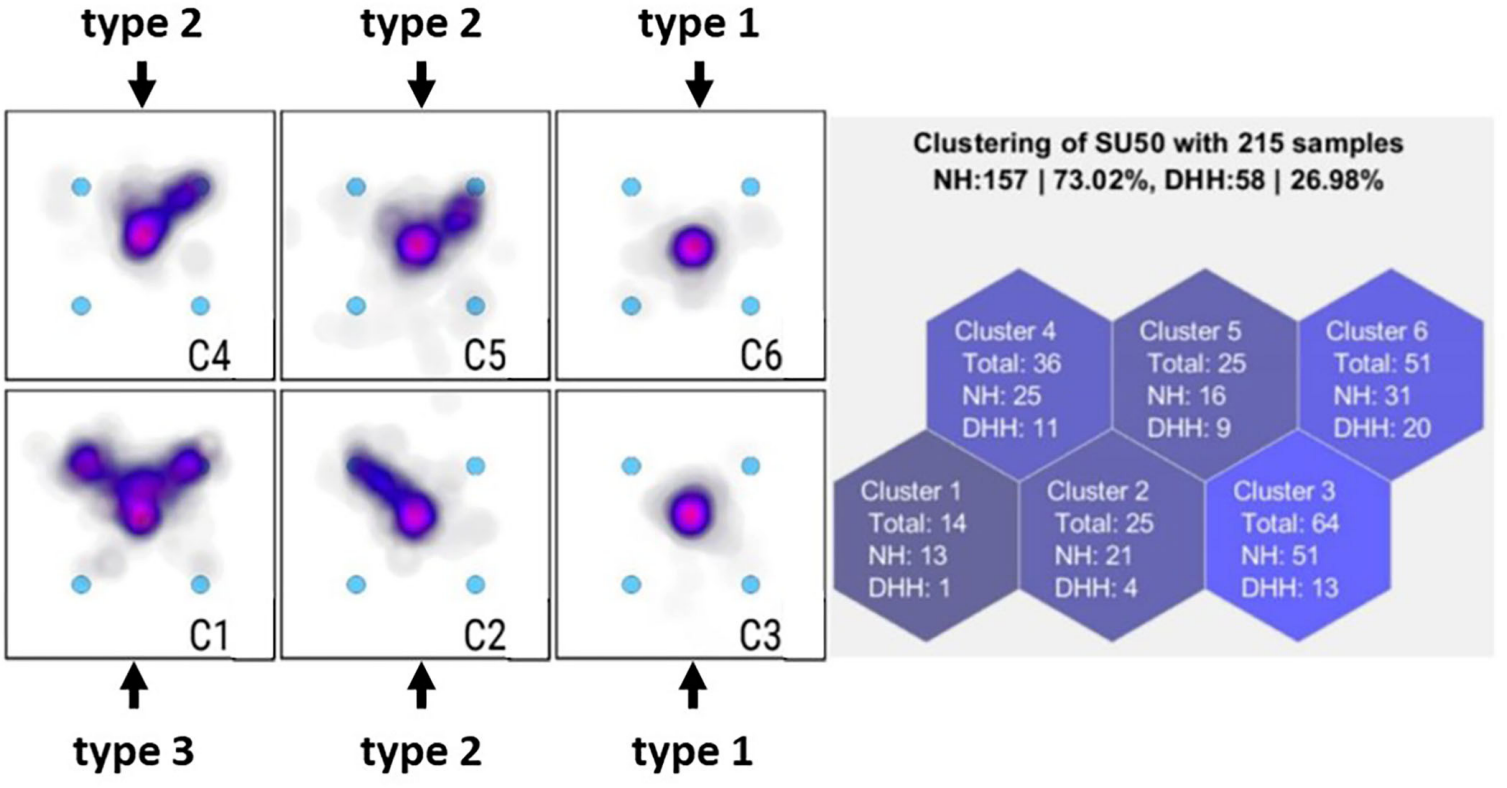
Example of clusters for the dice five (canonical arrangement, dice range).

The purpose of a clustering algorithm is to automatically assign multivariate data into subgroups (Everitt et al., 2011). In this study, each heatmap was grouped with similar heatmaps to automatically identify clusters, in which all heatmaps represent similar enumeration processes of the students (Figure 2.2). A cluster can be represented by its prototype, which, in this case, is the average heatmap (see Figure 3) of all the original heatmaps assigned to this cluster. Finding these groups automatically required two decisions by the system designer: First, the concept of (dis-) similarity for the data had to be defined. This relates to the question, “when are two heat maps similar to each other [see (1) in the following]?” Second, the specific clustering algorithm had to be chosen, and an assumption about the number of clusters or granularity of the data had to be made [see (2) in the following].

(1) Defining the similarity between two images is broadly explored in the digital image processing community (Goshtasby, 2012). While there is often the possibility to have domain- or application-specific definitions, we opted for a standard approach in this study and used the Euclidean distance *d* between two heatmaps as a measure for their dissimilarity. It was calculated by comparing two heatmaps *H*_1_ and *H*_2_ pixel by pixel, that is, summing the squared pixel differences:


$$\begin{array}{l} d={\left\|H_{1}-H_{2}\right\|}_{2}=\sqrt{\sum_{i=1}^{160}\sum_{j=1}^{90}{\left[H_{1}\left(i,j\right)-H_{2}(i,j)\right]}^{2}} \end{array}$$

Note that H_1_(i, j) describes the color of the pixel at position i, j in heatmap H_1_. The distance d for any two identical heatmaps is always zero, and the more the pixel values differ, the larger d becomes. Likewise, the pattern recognition and machine learning fields have spawned large numbers of different clustering algorithms (Everitt et al., 2011), all with different advantages and disadvantages of their own. As we are interested in explorative data analysis, this study applies the SOM algorithm (Kohonen, 2001). The distinctive feature of SOMs in comparison to other cluster algorithms is the *a priori* assumption of a grid structure defining a relationship between the clusters. This 2D-grid, from which the map part of the name is derived, can provide additional information for interpreting the clustering results. The algorithm contains an iterative optimization procedure, which randomly selects a heatmap and calculates the distance to all cluster prototypes. The closest prototype will then move toward the current heatmap, making the cluster prototype more similar to the heatmap. The moving cluster representatives' neighbors, as defined by the *a priori* grid, will also move in that direction, but to a lesser degree. Then, the next heatmap is chosen, and the procedure repeats until the optimization process converges, that is, the cluster centers stop moving when all similar heatmaps are assigned to the same cluster. Due to its nature, the algorithm may produce different results depending on the random initialization of cluster representatives, and the order the algorithm selects the individual heatmaps for comparison. For the algorithmic details, we refer to Kohonen (2001). For the implementation, we used the Matlab's Deep Learning Toolbox V. 13.0 (RRID: SCR_001622) with standard learning parameters.

(2) SOMs, like many other clustering algorithms, require choosing the number of groups and their relationships to each other as part of the algorithm. This can be done automatically by defining a cluster quality measure and performing an additional model selection optimization loop, which increases computational costs significantly, or it can be done manually by either guessing or choosing the number based on the domain knowledge. Since a previous study (Schindler et al., 2020b) identified three different processes, this study used six clusters in a 3 x 2 grid structure. Under the assumption that all the three different processes are present in the data and sufficiently dissimilar as measured by the Euclidian distance, they each form one group, and the additional groups can represent outliers, such as the previously unobserved processes or the subgroups of one of the three identified processes.

#### Cluster assignment

As a result of the clustering process, each heatmap is assigned to one of the six clusters (Figure 2C). The unused information in the clustering process can now be determined for each cluster. This includes the number of students who are DHH and hearing students and other statistics for each cluster.

#### Cluster prototypes

Since the clustering itself was performed on the size-reduced heatmaps, in the next step, full-size cluster prototypes (Figure 2.3) are calculated from the original heatmaps. To help with the visual inspection of the cluster prototypes and for easier distinction, we shift the color space for those prototypes from the red-green color table created by the ET software to a red-blue color table for the cluster prototypes. This can be done for RGB images by copying the green channel into the blue channel and then setting the green channel to zero (Figure 2D).

#### Qualitative analysis

The unsupervised learning results in six clusters each for every task (item) used in this study. For each of the non-empty clusters, we interpreted the average heatmaps (Figure 2.4). We assigned categories of processes based on a system that had been developed inductively in an earlier study (Schindler et al., 2020b). For example, for the dice patterns, we distinguished the following three *types of processes*:

*(1)    Simultaneous enumeration*: Presented dots are enumerated simultaneously. The gazes are predominantly in the middle of the pattern (on the middle point).
*(2)  Enumeration through the use of groups/structures*: Presented dots are enumerated through the use of groups/ structures: Gazes are on parts of the dots, for example, to one side, indicating the use of symmetries.
*(3)Enumeration through counting*: At least half of the dots are looked at—with gazes on at least half of the points—or every dot is counted, which means that gazes are on every dot.

Note that for the random arrangements in the counting range, the three types of processes were minimally different: (*1) Quasi-simultaneous enumeration, (2) Enumeration through the use of groups/structures*, and *(3) Enumeration through counting*. The first process in this case was the quasi-simultaneous enumeration because sets of dots in the counting range cannot be enumerated simultaneously but quasi-simultaneously (see Schindler et al., 2020b for more detail).

In practice, this means that, for the example of the dice five, we assigned type 1 to C3 and C6, type 2 to C2, C4, and C5, and type 3 to C1 (Figure 3).

Two of our researchers categorized all average heatmaps in this way. We assigned types of processes for each of the four conditions: (a) subitizing range (2–4) in random arrangements, (b) counting range (5–9) in random arrangements, (c) dice range (2–6) in canonical arrangements, and (d) beyond dice range (7–9) in canonical arrangements.

We then calculated how many student heatmaps were assigned to each type of process. For this, we drew on the information of how many heatmaps (of students who are DHH and of hearing students) were in each cluster. For example, for the dice five, we found (1) *type 1*: 33 (=13+20) DHH and 82 (=51+31) hearing, (2) *type 2*: 24 DHH and 62 hearing, and (3) *type 3*: 1 DHH and 13 hearing.

#### Statistical analysis

To identify group differences in student enumeration processes, we carried out k x 2 chi square tests, which allowed us to compare two independent samples for a k-stepped property (Figure 2.4). In our case, we had two groups (DHH, hearing) and three enumeration processes. We conducted a 3 x 2 chi square test for each condition: (a) subitizing range (2–4) in random arrangements, (b) counting range (5–9) in random arrangements, (c) dice range (2–6) in canonical arrangements, and (d) beyond dice range (7–9) in canonical arrangements. We calculated effect sizes using *Cramérs V*. According to Cohen (1988), *Cramérs V* can be interpreted as follows: *V* = 0.10 is a small effect, *V* = 0.30 is a medium effect, and *V* ≥ 0.50 is a large effect. For degrees of freedom of 2, like in our study, according to Cohen (1988) *V* = 0.07 is a small effect, *V* = 0.21 a medium effect, and ≥0.35 a large effect. Within the subitizing range, 1,961 items were analyzed (2–4 in three different arrangements); within the counting range, 2,972 items were analyzed (5–9 in three different arrangements); in the dice range, 1,084 items were analyzed (2–6 in one arrangement); and in the beyond dice range, 598 items were analyzed (7–9 in one arrangement). In the cases where the chi square test showed significant group differences, we calculated cell tests to investigate what the significant group differences were due to. We carried out the statistical analyses using the statistics and analysis software IBM SPSS 28 (RRID: SCR_019096).

## Results

**Figure 4:**
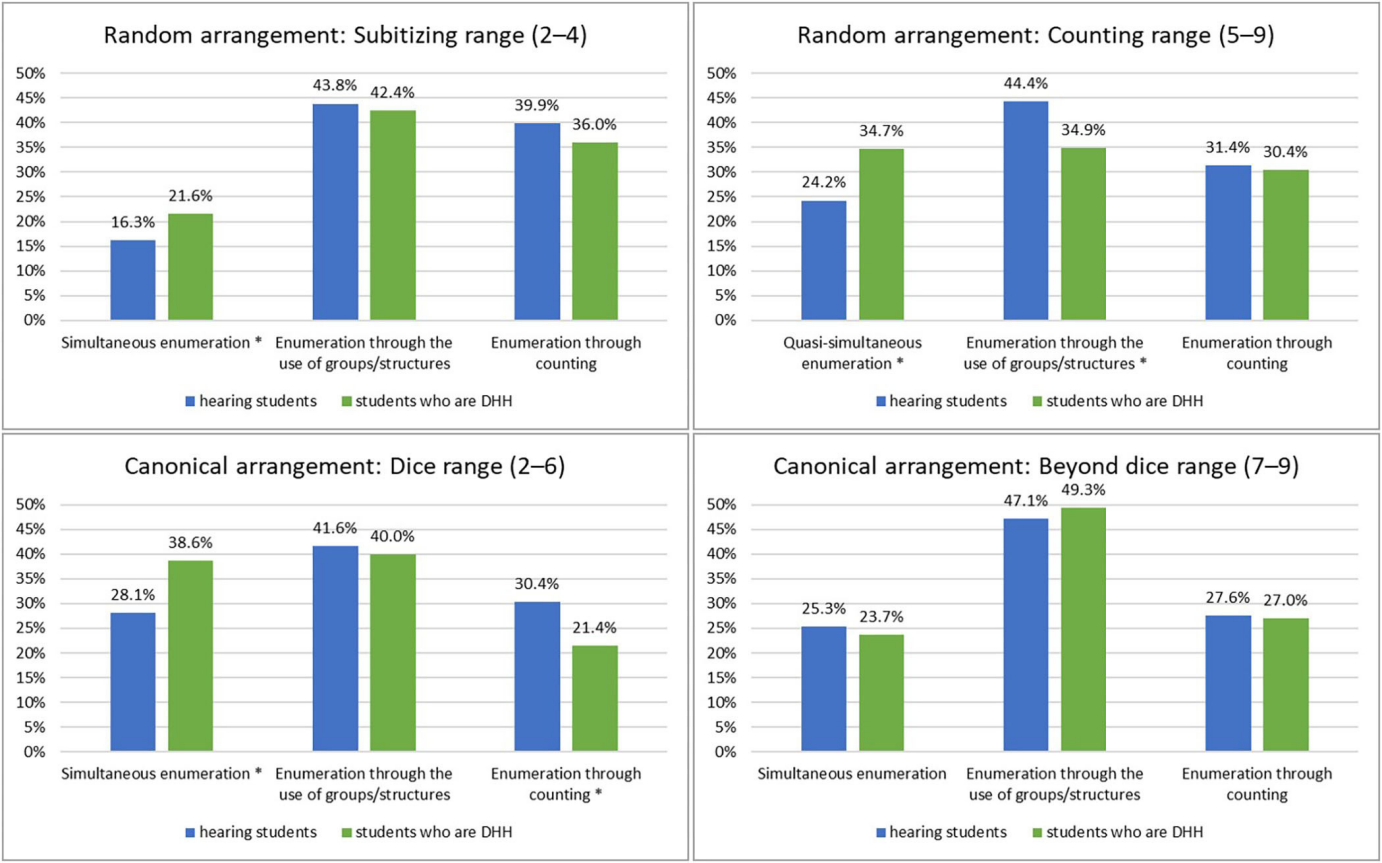
Distribution of enumeration processes in four conditions (significant group differences are marked with^*^).

Chi square tests were performed to investigate the relationship between hearing loss and enumeration processes. The chi square tests revealed significant differences^1^ in the distribution of enumeration processes (Figure 4) between students who are DHH and hearing students for both conditions in random arrangements: (a) subitizing range: χ^2^ (2) = 7.91, *p* = 0.038, *V* = 0.06 and (b) counting range: χ^2^ (2) = 35.54, *p* < 0.001, *V* = 0.11. Effect sizes were small. For the dice range in the canonical arrangement condition (c), chi square test also revealed significant differences with small effect sizes in the distribution of enumeration processes between DHH and hearing students: χ^2^ (2) = 13.87, *p* = 0.002, *V* = 0.11. Yet, no significant difference was found for the larger sets in canonical arrangement (beyond dice range): χ^2^ (2) = 0.26, *p* = 0.878. This means that the distribution of enumeration processes did not differ between students who are DHH and hearing students in the beyond dice range in the canonical arrangement condition. Cell tests for the differences between groups revealed the following results: *(a) Subitizing range:* Students who are DHH used simultaneous enumeration more often than hearing students (χ^2^ (1) = 7.53, *p* = 0.018, *V* = 0.06). *(b) Counting range:* Students who are DHH used quasi-simultaneous enumeration more often than hearing students (χ^2^ (1) = 31.74, *p* < 0.001, *V* = 0.10). Students who are DHH used enumeration of groups less often than hearing students (χ^2^ (1) = 20.95, *p* < 0.001, *V* = 0.08). *(c) Dice range in canonical arrangement:* Students who are DHH used simultaneous enumeration more often than hearing students (χ^2^ (1) = 11.04, *p* < 0.001, *V* = 0.10). Students who are DHH used enumeration through counting less often compared to hearing students (χ^2^ (1) = 8.49, *p* = 0.008, *V* = 0.09). Also here, all effect sizes were small.

## Summary and discussion

The aim of this study is to investigate if students who are DHH differ from hearing students in small number enumeration processes. We used the ET data of 227 students (164 hearing students, 63 students who were DHH). For analyzing the students' eye movements, we used ET in combination with AI, specifically a clustering algorithm, and the analysis of human experts to identify enumeration processes from the heatmaps of student gaze distributions. Our study investigates the enumeration processes of school-age students who are DHH in a large-scale study—and it brought interesting results to light, indicating that students who are DHH used more advantageous processes than their hearing peers in different task conditions. A further feature of this work is that we used AI to analyze the differences in small number enumeration processes between students who are DHH and hearing students. The ML-based processing pipeline introduced in this study, first and foremost, allowed us to efficiently process gaze heat maps for a large number of students by automatically clustering the enumeration strategies based on the patterns in the data. The automated clustering analysis also provided an independent view of the data that can support human researchers as an “AI colleague” in the interpretation of gaze data. The presented algorithms are not specific to the task of small set enumeration but can be applied to any set of heat maps. Hence, the pipeline can serve as a blueprint for similar studies, but also as a basis for the development of automated student assessment tools supporting researchers and teachers.

We examined student enumeration processes of sets under four conditions: both the subitizing range (2–4) and the counting range (5–9) in random arrangements, and both the dice range (2–6) and beyond dice range (7–9) in canonical arrangements. We found significant differences in the distribution of enumeration processes between students who are DHH and hearing students in both random arrangement conditions: In the subitizing range and the counting range, students who are DHH used more advantageous enumeration processes than hearing students. This means that students who are DHH enumerated the presented dots more often (quasi-) simultaneously, that is, they took in all dots at a glance. For dice patterns in the canonical arrangement, we also found significant differences in the distribution of enumeration processes between students who are DHH and hearing students. Also here, students who are DHH more often enumerated the presented items simultaneously, indicating that students who are DHH recognized and recalled the dice patterns more often than hearing students. However, it should be noted that the effect sizes were small for all significant differences; practically, the effect of group on students' enumeration processes is small. Yet, the significant differences are interesting against the backdrop of the following aspect: The sample of students who are DHH in our study was diverse, for example, regarding language use, which is known to be a major influencing factor on the learning and achievement of students who are DHH. Also, approximately two-thirds of the students who are DHH (65%) in our study had mathematical difficulties. With a large heterogeneity within this group in terms of language use and hearing loss and the fact that a substantial number of them had MD, it is remarkable that, still, significant group differences were found—with students who are DHH performing better than hearing students. Even if effect sizes are small, our findings indicate that students who are DHH in third to fifth grade do not appear to have difficulties in small number enumeration processes as compared to hearing students at that age but, rather, appear to use more advantageous enumeration processes than their hearing peers. They use subitizing and groupitizing more often, and they recognize and recall dice patterns more often. This is interesting and contributes to the state of research in different ways.

Bull et al. (2006) had shown that, for adults who are DHH and at a high academic performance level (university students), there are no differences in response times in small number enumeration as compared to their hearing peers. Our study showed that children who are DHH at the age of approximately 10–11 years not only perform as well as their hearing peers, but they partially appear to perform even better than their hearing peers in that specific area. This is interesting since also in our sample, the students who are DHH had significant difficulties in mathematics. These difficulties do not seem to go hand-in-hand with subitizing deficits—unlike students with MD, where a “dysfunctional subitizing mechanism” (Schleifer and Landerl, 2011, p. 280) appears to accompany MD. Our results indicate that the difficulty profile of students who are DHH appears to differ from that of students with MD in general.

Furthermore, our study found that the students who were DHH also performed better than their hearing peers in conceptual subitizing, also called groupitizing (Clements, 1999; Starkey and McCandliss, 2014): In the counting range, students who are DHH used the quasi-simultaneous enumeration more often, where it is necessary to use the part-whole relationship and where conceptual understanding is necessary (Krajewski and Schneider, 2009; Starkey and McCandliss, 2014). This is particularly interesting since students who are DHH generally perform lower than their hearing peers in mathematics. Still, our study indicates that they do have this fundamental conceptual knowledge and apply it even more often than their hearing peers.

Finally, it is worth mentioning that students who are DHH recognized and recalled dice patterns at one glance more often than their hearing peers. Less often, they needed to take in the dots serially. We can only speculate about the reasons. One possibility is that students who are DHH are more likely to memorize and recall visual patterns. Overall, it is often discussed that visual abilities are enhanced in relation to auditory deprivation in people who are DHH (Hauthal et al., 2013). Many studies have implied that a lack of auditory experience from an early age influences the organization of the human brain for peripheral and central visual processing and is often compensated with supranormal performance in other sensory systems, such as vision (Alencar et al., 2019). Scott et al. (2014) found that adults with profound, congenital, and hereditary hearing loss have a network of brain regions exhibiting enhanced responsiveness to peripheral visual stimuli. Furthermore, deaf persons appear to have a different visual viewing behavior in some aspects, for example, a preferential central fixation pattern compared to hearing persons (Lao et al., 2017). However, this link to a better visual ability and performance has predominantly been associated with sign language, as some advantages in visual-spatial tasks are not only found in deaf individuals but also in hearing individuals who are skilled signers (Marschark et al., 2015). However, in our study group, most of the students who were DHH used spoken language or sign-supported speech as a preferred communication method, not sign language. Yet, in their regular schooling, they were in contact with sign language frequently, and it is further possible that visual orientation played an important adaptive or compensational role for these students, as well as in their teaching. Didactics and teaching materials in the special schools for students who are DHH are often supported by visualizations in such a way that they can be easily accessed by all students, both sign language and spoken language users (Nunes, 2004). Knoors and Marschark (2014) emphasized in their research that students who are DHH are not hearing learners who simply cannot hear; they may utilize different abilities in dealing with tasks.

Only in the canonical arrangements beyond the dice range did the students who are DHH and the hearing students apply processes similarly. One possible explanation is that here, the hearing students had more experience dealing with symmetries, and this compensated for the advantages that students who are DHH may have had otherwise.

## Limitations and implications for future studies

Besides summarizing and discussing our results, we want to discuss the *limitations* of our study and point out some suggestions for *future work* that emerge. One possible limitation of this study lies in the selection and composition of the sample. The group of hearing students consisted of fifth-grade students (mean: 10.9 years, SD: 0.7 years), and the group of students who are DHH consisted of fifth-, fourth-, and third-grade students (mean: 11.9 years, SD: 1.0). So, students who are DHH in our study were on average in lower grade levels, but at the same time older than the hearing students, which makes it difficult to compare the groups in relation to their mathematical development in general. However, given that children learn to enumerate small numbers of dots well before the age of 10—before or at the very beginning of school—and since the curriculum in grades 3–5 does not cover small number enumeration, the grade levels were not critical for this skill. Therefore, the heterogeneity of the groups was acceptable and—in our view—biased the results marginally at most. Furthermore, the group of students who are DHH showed a large heterogeneity in terms of familial first language (L1), their communication modality, the degree of hearing loss, and their use of hearing aids or cochlear implants. On the other hand, the composition of the group of students who are DHH in our study represented well the heterogeneity of students who are DHH that is often encountered in special schools for students who are DHH in Germany: The DHH sample in our study had the commonality that they attended special schools for students who are DHH in Germany and they all had a peripheral hearing loss and, in a few cases, a central auditory processing disorder, yet, they were diverse otherwise. Subgroups were small, which prevented us from running subgroup tests. However, for future research, it would be valuable to look closer into language abilities and group differences within the group of students who are DHH in terms of communication modality, degrees of hearing loss, etc.

Regarding the applied clustering methodology, it is worth noting that the generic approach we used in this article was not optimized for this application. While we do not expect substantially different results if a different clustering algorithm had been applied, the choice of the right dissimilarity measure might be more sensitive in the future. In this study, we used the Euclidean distance, which may not be optimal in cases where the appearance of the heatmaps that belong to one enumeration process is, in part, the same as the appearance of the heatmaps belonging to another process. In such cases, the heatmaps that correspond to two different processes will be scored to be similar and might end up in the same cluster. Hence, investigating distance metric learning methods for eye-tracking heatmaps that provide a stronger separation for those cases is a promising topic to investigate in the future (see Xing et al., 2002).

## Conclusion

The results of this study indicate that students who are DHH do not lag in the enumeration of small numbers compared to hearing students but rather appear to use more advantageous processes in enumerating small sets than their hearing peers. Our study suggests that difficulties in enumerating small numbers do not coincide with the MD that students who are DHH often have, which offers interesting perspectives for further research.

## Pedagogical implications

For educational practice, our results indicate that students who are DHH—regardless of their language modality—appear not to need support in enumeration processes, such as the part-whole relationship. In this respect, the need for support differs from that necessary for students with MD in general, where the practice of, for example, basic visual small number enumeration processes can be valuable, especially in early grades. In our study, even students who were DHH and had MD showed advanced skills in small number enumeration. For supporting students who are DHH, a focus on other aspects of mathematics learning that are known to be difficult for DHH learners, including conceptual understanding, problem-solving, and word problem solving, appear to be more significant. These results and consequences are to be taken into consideration for teacher training in special education, ideally across all spoken and signed languages, which should be guided by the needs and strengths of the group of students who are DHH. Some pedagogical suggestions for DHH teachers and early intervention practitioners “from research to practice” have been recently made; for example, on teaching numeracy and early math concepts (Kritzer and Green, 2021), fostering fraction learning (Mousley, 2021), spatial reasoning (Thom and Hallenbeck, 2021), and usage of sign language in the mathematics classroom (Krause and Wille, 2021). Our study indicates that small number enumeration appears to be among the relative strengths of students who are DHH. This is encouraging given the general difficulties and delays in their mathematical development.

## Data availability statement

The raw data supporting the conclusions of this article will be made available by the authors, without undue reservation.

## Ethics statement

Ethical review and approval was not required for the study on human participants in accordance with the local legislation and institutional requirements. Written informed consent to participate in this study was provided by the participants' legal guardian/next of kin.

## Author contributions

MS and KS contributed to the conception and design of the study. MS, JD, and AS conducted the study and collected the data. MS, JD, AS, ES, and AL performed the data analysis. MS, AS, ES, AL, and KS wrote the manuscript. All authors read and approved the submitted version.

## Conflict of interest

The authors declare that the research was conducted in the absence of any commercial or financial relationships that could be construed as a potential conflict of interest.

## Publisher's note

All claims expressed in this article are solely those of the authors and do not necessarily represent those of their affiliated organizations, or those of the publisher, the editors and the reviewers. Any product that may be evaluated in this article, or claim that may be made by its manufacturer, is not guaranteed or endorsed by the publisher.

## Footnotes

^1^Bonferroni-Holm adjusted *p*-values are given in the following.
